# Remarks Concerning Some of the Diseases Prevailing among the Freedpeople in the District of Columbia

**Published:** 1866-08

**Authors:** Robert Reyburn

**Affiliations:** Surgeon, U.S. Vols.


### REMARKS CONCERNING SOME OF THE DISEASES PREVAILING AMONG THE FREEDPEOPLE IN THE DISTRICT OF COLUMBIA.

(Bureau Refugees, Freedmen, Abandoned Lands.)

By ROBERT REYBURN, Surgeon, U.S. Vols.

The appended Report, which gives the results of the treatment of seven thousand nine hundred and forty-nine (7,949) cases of sick and wounded freedmen, who were under medical treatment in District of Columbia, Bureau of Refugees, Freedmen, and Abandoned Lands, from June 1, 1865, to December 31, 1865, has developed some interesting facts relative to the diseases prevalent among them, to which we desire to call the attention of the medical profession.

This report does not embrace *all* who were under treatment during the above months, but only those cases that were recorded and the results known.

This District Bureau of R. F. and A. L. embraces the city of Washington and the District of Columbia, properly so-called, the three counties of Alexandria, Fairfax, and London, Va., and a part of St. Mary's Co., Md., together with a general supervision of freedmen's affairs in the whole State of Maryland. The total number of freedpeople who are more immediately under the charge of the Medical Department of this district, is, from a census taken by order of the Bureau, and which is now nearly completed, about fifty thousand; of these somewhat over fifty-two per cent. are full blood Africans, and the remaining forty-eight per cent belong to the mixed races.

The next and last class of diseases to which we will here advert is the tubercular; this includes consumptiom, what is usually denominated scrofula, and all the other varied forms in which the tubercular diathesis manifests itself.

Out of the total number, as above stated, three hundred and forty-six deaths are recorded, being about forty-three per thousand of those under treatment. This mortality is larger than exists at the present time in this district, and arises from the fact that the greater number of these cases were treated in hospital, and as only the more severe class of cases are sent to hospital, the mortality is larger than the average mortality of the cases treated in hospital and at their homes. Our mortality has been for the last three months about thirty-three per thousand of the number of the cases under treatment, and is now diminishing rather than increasing in amount.

The first and most fatal class of cases (although not the most numerous) in the report is that of typhoid fever. The number of cases of this disease was one hundred and sixteen, and the number of deaths was forty-nine, or nearly forty-one per cent of the number of cases treated. The *post mortem* examinations made in the fatal cases presented the usual abdominal lesions characteristic of typhoid fever, and the only point of difference observable was that the pulmonary and cerebral inflammations which so frequently complicate this disease, and add to its dangers, were found to present a more asthenic type, and consequently were found to be more dangerous in their results than is commonly the case with typhoid fever occurring among whites. The pneumonia which we find so often in cases of typhoid fever occurring in the Caucasian race, in the negro becomes pleuro-pneumonia of a low grade, and is generally accompanied with a large amount of serous or sero-sanguineous fluid, which, after death, is found filling the cavities of the pleuræ. In fact, as a rule, inflammations of the serous membranes seem to be much more frequent and dangerous among negroes than whites, and it is rarely we have examined a negro cadaver which did not present evidences of recent serous inflammations in either the thorax, abdomen, or calvarium. The same condition above mentioned has been found by other observers, and we have been informed by Dr. Ira Russell (late Brevet Lieut-Col. and Surgeon, U.S. Vols.) that he has hardly ever opened a negro cadaver in which there was not a large amount of serous exudation. The observations of Dr. Russell on this point are deserving of especial weight on account of his having made the diseases and pathology of the negro race a specialty, and from his having assisted in making a large number of *post mortems* of freedpeople, while on duty in the West during the war. It may, however, be objected that in some of the above cases the effusion may have been *post mortem*, but we feel convinced that such was not the case, for the following reasons, *viz.*:—

1st. In many of these cases, percussion revealed the existence of the effusion during life; and, secondly, the amount of effusion was generally so great as to entirely preclude the idea of accounting for it by any mechanical transudation of the serum of the blood through the walls of the bloodvessels after death.

The next class of diseases worthy of notice, is that comprising the various forms of remittent and intermittent fevers. The total number of cases belonging to this class was two thousand seven hundred and seventy-six, or about thirty-five per cent of the total under treatment. This significant fact is, we believe, a sufficient answer to, and refutation of, the statement so often reiterated in our text-books, that the negro race are not subject to, and do not suffer from, malarious diseases.

Now, it may be that in Africa and the West Indies they do not suffer to the same extent that unacclimated whites do, but they certainly are not exempt from these diseases in this country; and, as far as our own opinion goes, we are strongly inclined to the belief that this so-called exemption has no foundation in fact, and is unworthy of credence.

In order to show that the large number of cases of intermittent and remittent fevers occurring among the freedpeople of this district is not owing to the influence of temporary or local causes, we are permitted, through the courtesy of Surgeon C. W. Hornor, U.S. Vols,, Chief Medical Officer of Bureau Refugees, Freedmen, and Abandoned Lands, to give a few extracts fram the official reports received from other districts of this Bureau, which will, we think, confirm and strengthen our assertions.

The official monthly report for September, 1865, of the Surgeon-in-Chief of the District of South Carolina (Bureau R. F. and A. L.) for the cities of Charlestown, Beaufort, and Combahee Ferry, and St. Helena Island, is now before us, and from it we extract the following:—

"The total number of sick freedpeople under treatment during the month was one thousand and ninety-six, and the number of cases of the above-mentioned diseases was three hundred and ninety-seven, or about forty per cent of the total under treatment."

From a like official report for October, 1865, from the District of Virginia, Bureau R. F. and A. L., for the cities of Yorktown, Petersburg, Hampton, and Eastville, we find the following figures, *viz:*—

"The total number of sick freedmen under treatment during the month, was seven hundred and eighty-eight, and the number of cases of malarious diseases was four hundred and forty-one, or nearly fifty-six per cent of the total number under treatment."

From a similar report for the month of October, 1865, from the District of Georgia, Bureau R. F. and A. L., and for the cities of Macon, Savannah, and Augusta, we extract the following:—

"The number of sick freedmen under treatment during the month was five hundred and eleven, and the number of cases of the above diseases was one hundred and sixty-seven, or nearly thirty-three per cent of the total number under treatment."

We might continue to make extracts similar to the above to an indefinite extent, but we think it to be unnecessary, as all the reports we have seen, and we have consulted a large number with especial reference to this point, give uniformly the same result.

The total number of cases belonging to this class was two hundred and ninety, or nearly four per cent. of the total number of cases treated. This result is somewhat striking, and is diametrically at variance with the extraordinary statements which have been made by some of our medical authors and teachers, and which are to the effect that scrofula (in some form) is almost universally prevalent among the negroes and mulattoes in northern latitudes.

Now, is it undoubtedly true that when the negroes and mixed races are exposed for a lengthened period to the combined evil influences of improper nourishment, insufficient clothing, impure air, and uncleanliness, that they are the victims of the various forms of scrofulous disease; but where well fed and clothed, and subjected to favorable hygienic influences, we are fully persuaded that such is not the case; and we believe that the statements above referred to are entirely mistaken and erroneous. As a proof of this we may cite the fact, that the same causes which produce and perpetuate scrofula among the colored people, may be seen (to a limited extent) in operation among the poorer classes of white people in many of the larger cities of the Union, and produce among them precisely similar results; in other words, we believe that scrofula is nothing more than the effect produced upon the human system by unfavorable hygienic influences, and any of the races of men will suffer from it in exact proportion as they are subjected to these deleterious conditions.

In this District Bureau of R. F. and A. L. there are now three hospitals open, and eleven physicians employed by the Bureau, who attend them both at the hospitals and at their homes. The number of patients under treatment during each month ranged from eighteen hundred to two thousand for some months past, and if scrofula occurred as frequently among them as has been represented, we think we would have had fair opportunities for observing it. There are many other points of interest connected with the diseases of the freedpeople, on which we might dwell, but the amount of space we have allotted to ourselves in this hasty sketch will not permit us to do so at this time.

